# Shared understanding and social connection: Integrating approaches from social psychology, social network analysis, and neuroscience

**DOI:** 10.1111/spc3.12710

**Published:** 2022-10-17

**Authors:** Elisa C. Baek, Carolyn Parkinson

**Affiliations:** ^1^ Department of Psychology University of California Los Angeles California USA; ^2^ Brain Research Institute University of California Los Angeles California USA

**Keywords:** fMRI, information sharing, shared reality, social connection, social neuroscience, social networks

## Abstract

Meaningfully connecting with others is critical to the well‐being of individuals. What phenomena contribute to and stem from social connection? In this paper, we integrate emerging work that uses neuroimaging and social network analysis with theories that explore the links between shared reality and social connection. We highlight recent work suggesting that the extent to which people have aligned mental processing and shared subjective construals to those around them—as shown by neural similarity—is associated with both objective and subjective social connection. On the other hand, idiosyncrasies are linked to difficulties with social connection. We conclude by suggesting how the links between shared understanding and social connection can be productively used as a framework to study psychosocial phenomena of interest.

## 1 INTRODUCTION

Humans are inherently social, with a fundamental need to belong and forge meaningful connections with others (Baumeister & Leary, 1995). Indeed, social connection is critical to individuals' well‐being, and deficits in social connection can have devastating consequences, including increased risk for mortality that persists even after controlling for comorbidities (Cacioppo & Cacioppo, 2014; Hawkley & Cacioppo, 2010; Hawkley et al., 2003; Moieni & Eisenberger, 2020).

A rich body of research highlights that experiencing generalized shared reality with others plays an important role in achieving social connection (Baumeister et al., 2018; Echterhoff & Higgins, 2018; Higgins et al., 2021; Reis et al., 2000, 2017; Rossignac‐Milon & Higgins, 2018). For instance, feeling understood by others is associated with positive evaluations of social interactions with strangers (Cross et al., 2000), greater fulfillment in close relationships (Oishi et al., 2010), and increased life satisfaction (Lun et al., 2008; Reis et al., 2000). Furthermore, the pursuit of generalized shared reality with others—which refers to the perceived experience of having similar inner states (e.g., beliefs, feelings, and attitudes) about the world in general to those of others (Echterhoff et al., 2009; Higgins et al., 2021)—is closely linked to the pursuit of social connection, as the pursuit of generalized shared reality is driven not only by people's motivations to understand the world around them (“epistemic motives”; Higgins et al., 2021) but also by their motivations to relate to one another interpersonally (“relational motives”; Higgins et al., 2021). Indeed, people seek to achieve generalized shared reality not only with close others, but also strangers, and the extent to which people achieve generalized shared reality is associated with success in attaining social connection (Rossignac‐Milon et al., 2021).

In the current paper, we integrate findings from a growing body of literature that uses theories, tools, and methods from social network analysis and neuroscience with existing theories that link generalized shared reality with social connection. In doing so, we highlight that neuroimaging work corroborates established links between generalized shared reality and social connection, while also providing additional insights on what aspects of mental processing of what kinds of stimuli may be particularly important. We posit that experiencing shared understanding across various contexts (e.g., of multiple events or situations) may contribute to and stem from generalized shared reality (i.e., a more general, subjective sense of experiencing common inner states with others about the world broadly). We further highlight potential mechanisms that may underlie this bidirectional relationship. We also review evidence that suggests that our motivations to connect with one another through cultivating generalized shared reality constitute one factor that drives information sharing, a ubiquitous and consequential behavior.

## 2 NEURAL SIMILARITY AS A MEASURE OF SHARED UNDERSTANDING

We begin by providing an overview of how neuroimaging can be used to measure similarities in mental processing related to subjective construals across various contexts, thereby capturing shared understanding while individuals view and interpret stimuli. Specifically, measuring brain responses in naturalistic paradigms (e.g., where participants view audiovisual stimuli, such as videos, that unfold over time) can unobtrusively provide insight into participants' mental processes that ebb and flow over time in response to stimuli that mimic the multisensory and dynamic nature, as well as the contextual richness, of everyday experiences. One can then correlate these brain responses across participants to obtain intersubject correlations (ISCs), which measure the degree to which participants show similarities in brain responses and thus, can indirectly capture intersubject similarities in mental processes associated with particular brain regions (e.g., attention allocation, interpretations, emotional responding) while processing stimuli (Nastase et al., 2019). Indeed, coordinated neural responses across individuals (i.e., large ISCs of brain responses) have been linked to shared understanding of events (Lahnakoski et al., 2014; Nguyen et al., 2019; Yeshurun et al., 2017); participants who had similar interpretations of an animated narrative, as indicated by linguistic similarities during free recall, also had greater neural similarity in brain regions implicated in high‐level cognition (Nguyen et al., 2019), and similarities in experimentally‐manipulated psychological perspectives during an audio narrative were also associated with similarities in neural responses in brain regions that support attentional allocation and subjective interpretation of events (Lahnakoski et al., 2014; Yeshurun et al., 2017). Combined, these findings support the notion that neural similarity can capture the extent to which people experience shared understanding with one another across various contexts.

Calculating similarities in neural responses as participants view naturalistic stimuli can garner insight into psychological processes that may not be easily captured with self‐report measures alone. For instance, whereas self‐report measures typically capture participants' responses at specific moments, ISCs capture similarities in how responses evolve over time across individuals, without requiring individuals to pause for introspection (Nastase et al., 2019). Additionally, ISCs can unobtrusively capture similarities in many different types of mental processing in parallel (e.g., emotional and socio‐cognitive processes) as they unfold without the need for participants to pause and self‐reflect on each aspect of mental processing that experimenters have predetermined to be of interest in order to report on it. Furthermore, not relying solely on self‐report can be advantageous because people are often unwilling and/or unable to accurately reflect on their cognitive processes (Nisbett et al., 1977) and because the act of self‐reflection can produce inaccuracies in reporting (Wilson et al., 1993; Wilson & Schooler, 1991). As we discuss below, researchers can leverage these advantages of neuroimaging to identify hypotheses about psychological processes that can be more directly tested with complementary self‐report and behavioral measures. In these ways, neuroimaging can be used synergistically with traditional behavioral and experimental methods in social psychology to study links between shared reality and social connection. In the following sections, we highlight neuroimaging research that corroborates these established links, showing that shared understanding of various stimuli—as captured by similar neural responses across individuals—is associated with both objective and subjective social connection. We also suggest that neuroimaging can add insight to the *types* of mental processing (e.g., what aspects of their world people pay attention to, how and when they deploy social cognitive processes like mentalizing) that may be particularly important in linking shared reality and social connection, which can then be more directly tested in follow‐up behavioral experiments.

### 2.1 Shared understanding is important to objective and subjective social connection

#### 2.1.1 Evidence from investigations of social connection in dyadic contexts

**FIGURE 1:**
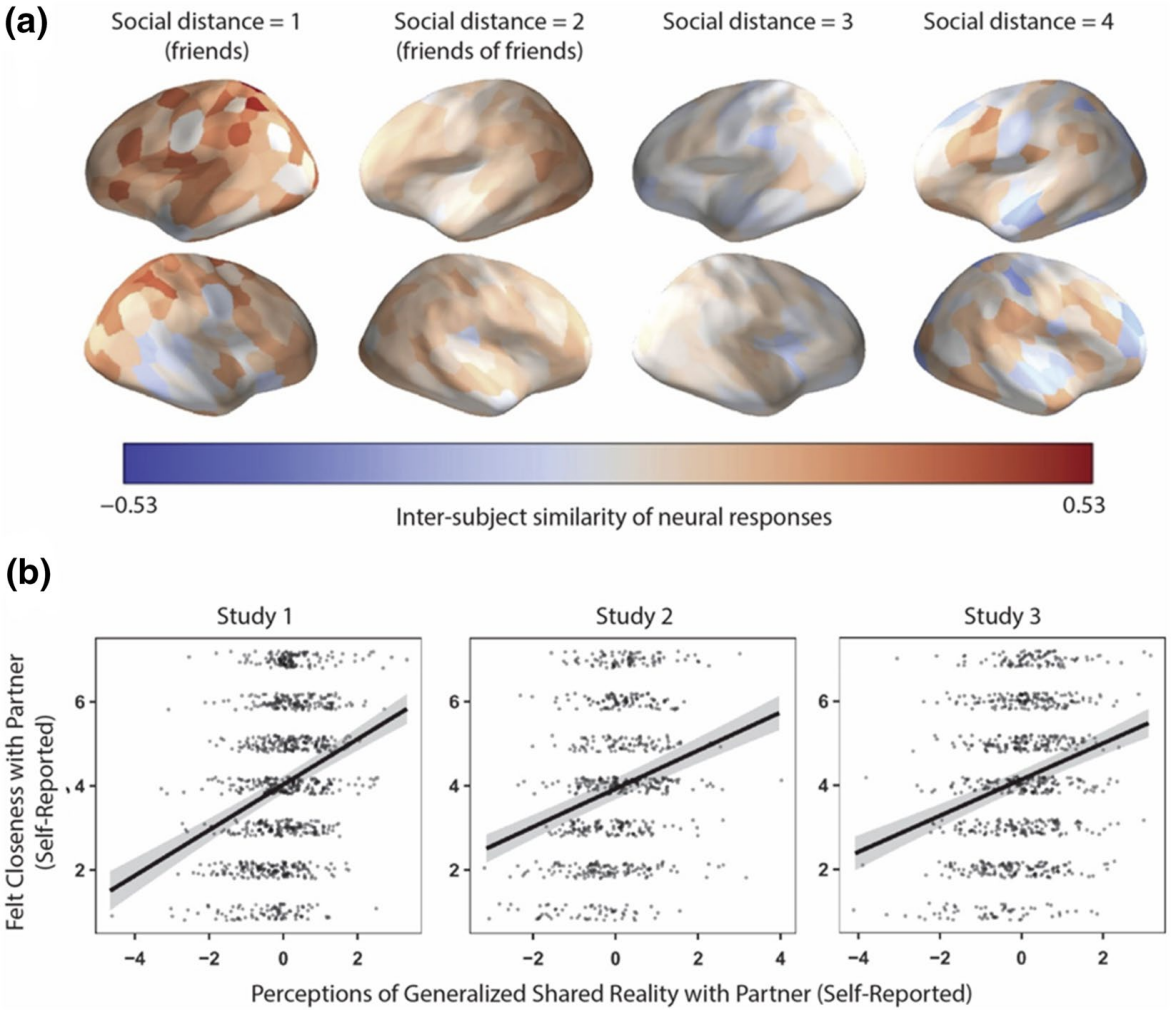
Social connection is linked to aligned mental processing and the perception of generalized shared reality in dyads. (a) Inter‐subject similarity of neural responses to naturalistic stimuli (e.g., film clips), which can capture alignment in mental processing, is associated with social distance; friends show more similar neural responses to one another compared to people further apart in the social network. *Source*: Redrawn from Hyon et al. (2020). (b) Across three studies, self‐reported perceptions of generalized shared reality with a partner were associated with felt closeness with the partner. *Source*: Redrawn from Rossignac‐Milon et al. (2021)

Across diverse interpersonal contexts, experiencing shared understanding with others about various facets of the world has been associated with social connection (Andersen & Przybylinski, 2018; Bar‐Shachar & Bar‐Kalifa, 2021; Higgins et al., 2021; Rossignac‐Milon et al., 2021). Having many incidences of shared understanding can lead to a sense of generalized shared reality, or a broader perception that one shares their inner states with another person about the world in general (Echterhoff & Higgins, 2018; Higgins et al., 2021). Indeed, the homophily principle is pervasive in social networks, such that “birds of a feather flock together”—people tend to be surrounded by and be friends with individuals who are similar to themselves in demographic attributes such as age, religion, ethnicity, gender, and socioeconomic status (McPherson et al., 2002), as well as stable characteristics such as personality traits (Youyou et al., 2017) and personal values (Byrne, 1961; Dehghani et al., 2016; Lönnqvist & Itkonen, 2016; Youyou et al., 2017). Decades of research in social psychology has also highlighted the similarity‐attraction relationship: people are attracted to others who share similar attitudes (Byrne, 1961, 1997; Hyon et al., 2020; Montoya & Horton, 2013). Relatedly, similarities in how individuals construe the world are associated with social connection; in both strangers and romantic couples, dyads who experience a greater sense of generalized shared reality also experience greater interpersonal connection (Rossignac‐Milon et al., 2021; see Figure 1).

Neural evidence converges with and adds additional insight to the findings summarized above. As discussed previously, obtaining similarities in neural responses across individuals can be valuable in capturing alignment of mental processes between individuals. Brain‐to‐brain synchrony has been studied in various ways, including by exposing individuals to the same time‐locked stimuli (e.g., video clips; Hyon et al., 2020; Parkinson et al., 2018) and by examining unconstrained dyadic interactions (e.g., during live conversation; Kinreich et al., 2017). Recent work using these methods suggests that similarities in neural responding across individuals are related to social connection. For example, friends have similar neural responses to one another while viewing naturalistic videos (e.g., excerpts from film and television shows ranging in tone and topic), including in brain regions that govern attentional allocation, narrative interpretation, and affective responding (Hyon et al., 2020; Parkinson et al., 2018; see Figure 1). These neural findings not only support the notion that having similar construals of aspects of the external world is associated with social connection, but also provide insight into the *types* of similarities in mental processing that may be particularly related to social connection; for instance, given that similarity in brain regions implicated attentional allocation is associated with friendship, being similar in what you attend in the world around you may be particularly important in friendships. In another study, brain‐to‐brain synchrony during naturalistic, dyadic social interactions (i.e., conversations) was correlated with behavioral markers of social connection (e.g., shared gaze and coupling of positive affect; Kinreich et al., 2017); thus, biological coupling between individuals may be one indicator of shared subjective responding that is associated with social connection. Furthermore, people—often without conscious effort—imitate one another's behaviors, and this behavioral synchronization may both promote and reflect generalized shared reality, which is linked to social connection (Echterhoff & Higgins, 2018; Higgins et al., 2021). For instance, both non‐verbal and verbal mimicry are linked to social connection, such that individuals non‐consciously mimic the behaviors of interaction partners when trying to affiliate (Lakin & Chartrand, 2003) and verbal mimicry is associated with cohesiveness in teams (Gonzales et al., 2010), stability in romantic relationships (Ireland et al., 2011), and quality of social interactions (Niederhoffer & Pennebaker, 2002).

#### 2.1.2 Evidence from investigations of individual differences in overall social connection

Beyond particular dyadic interactions and relationships considered in isolation, the overall degree to which someone achieves shared understanding with others across various contexts may also relate to how successful they are in cultivating and maintaining social connection, and whether or not they occupy positions in their social networks that are conducive to social connection. For instance, “social chameleons”–people who are especially attuned to social cues and adapt their behaviors to meet others' expectations in social contexts (i.e., individuals high in self‐monitoring; Snyder, 1974)–are more likely to occupy social‐network positions where they act as brokers between otherwise unconnected people (Fang et al., 2015; Kleinbaum et al., 2015; Oh & Kilduff, 2008). Such individuals tend to be more successful in professions that require interacting with others (Baek & Falk, 2018; Kilduff & Day, 1994; Wang et al., 2015), perhaps because they are adept at achieving generalized shared reality, and/or perceptions thereof, with many others (even if they are pretending and do not *actually* see the world similarly to others).

**FIGURE 2:**
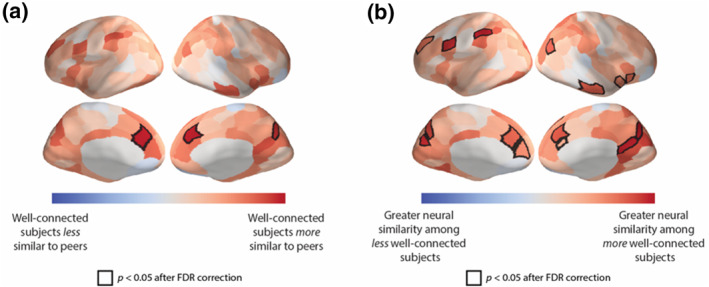
Overall degree of social connection in a community is associated with neural similarity to other community members. (a) Well‐connected individuals (e.g., whom many others indicated as a friend) in the social network of a residential community showed neural responses that were, on average, more similar to other community members compared to less well‐connected individuals. *Source*: Redrawn from Baek et al. (2022). (b) The findings followed an Anna Karenina principle, such that well‐connected individuals were exceptionally similar to one another, whereas less well‐connected individuals were dissimilar to each other, reflecting that each less well‐connected individual was dissimilar in their own way. *Source*: Redrawn from Baek et al. (2022)

Recent neuroimaging work also suggests that convergent processing with one's peers is associated with both objective and subjective measures of one's overall level of social connection, whereas idiosyncrasy is associated with overall social disconnection (Baek et al., 2022; Baek, Hyon et al., 2021). For instance, in one study, individuals with high levels of objective social connection (e.g., who many indicated as a friend and were therefore well‐connected in their social networks) showed neural responses more similar to normative neural responses in their communities compared to individuals with low levels of objective social connection (i.e., who were less well‐connected) while viewing naturalistic audiovisual stimuli (e.g., clips from television shows and movies) (Baek et al., 2022; see Figure 2). Additionally, these findings followed an Anna Karenina principle, which is based on the famous line from the novel *Anna Karenina*: “Happy families are all alike; every unhappy family is unhappy in its own way” (Tolstoy, 1878). Accordingly, well‐connected individuals had very similar neural responses to one another, whereas each less well‐connected individual was dissimilar in their own way (Baek et al., 2022; see Figure 2), suggesting that well‐connected individuals process the world similarly to one another, whereas each less well‐connected individual processes the world in their own idiosyncratic way. Notably, while self‐reported ratings of the content followed similar patterns, such that well‐connected individuals were also more similar to their peers in what they found to be enjoyable and interesting, controlling for these ratings did not change the neural results (Baek et al., 2022). Accordingly, these findings highlight advantages of neuroimaging, suggesting that neural data can capture aspects of mental processing beyond what can be obtained using a few targeted self‐report measures. For instance, brain areas where well‐connected individuals showed, on average, greater similarity with community members included regions that have been previously implicated in social cognitive processes such as understanding others' mental states (i.e., mentalizing; Baek et al., 2022). In these ways, neural data can provide insight into the types of similarities in mental processing that may be particularly strongly linked to social connection (e.g., similarities in when, and to what extent, people deploy social processing, such as mentalizing) that can then be tested in follow‐up studies more directly with, for instance, behavioral measures (e.g., to explicitly test if alignment in social processing is associated with social connection). Combined, these findings show that convergent processing of the world with one's peers is associated with *objective* social connection, or an individual's number of social ties, and highlight how neuroimaging can complement and extend insights from self‐report measures.

Thus, shared understanding of various stimuli is linked to *objective* social connection. However, it is also important to consider *subjective* social connection, given that one may have many friends, but feel very isolated, and vice versa; moreover, one's subjective feelings of social isolation can be even more consequential than objective social isolation (e.g., for negative health outcomes; Holwerda et al., 2014; Lee & Ko, 2018). Recent work highlights that similar patterns to those described above were found when investigating *subjective* social connection, or the distressing feeling that often accompanies subjective perceptions of social disconnection (i.e., loneliness) (Baek, Hyon et al., 2021). While non‐lonely individuals were highly similar to one another in their neural responses while viewing clips from television shows and film, highly‐lonely individuals were dissimilar not only to their non‐lonely peers but also to each other, highlighting that subjective social disconnection is associated with idiosyncratic processing, or a lack of shared understanding of external stimuli, which may also be associated with a lack of generalized shared reality more broadly (Baek, Hyon et al., 2021). Notably, these findings persisted after controlling for friendships between the participants, as well as overall levels of *objective* social connection (in this case, number of friends), suggesting that being surrounded by people who see the world differently from oneself (i.e., lack of generalized shared reality) may be a risk factor for loneliness, even if one regularly socializes with them.

Brain regions where similarities in neural responding have been associated with people's overall levels of objective and subjective social connection include regions in the default mode network, where neural similarity has been associated with shared understanding across various contexts (e.g., shared perspectives and goals; Lahnakoski et al., 2014; similar interpretations of ambiguous narratives; Nguyen et al., 2019; similar beliefs about characters; Yeshurun et al., 2017) and friendship (Parkinson et al., 2018). These findings corroborate recent theories suggesting that the default mode network plays a dynamic, sense‐making role in integrating individuals' internal schemas and memories with external information to form models of situations as they unfold, and that this integrating role of the network plays a critical role in establishing generalized shared reality (Yeshurun et al., 2021). Indeed, across multiple studies, synchronized neural responses between caregivers and children in regions of the default mode network were observed during active interaction (Kinreich et al., 2017; Piazza et al., 2020), supporting the notion that these regions in the brain support the creation of generalized shared reality across individuals. Combined, these results highlight that the degree to which individuals experience shared understanding across various contexts—and thus, generalized shared reality—with others in their social circles is critical to achieving social connection.

## 3 MECHANISMS THAT LINK SHARED UNDERSTANDING AND SOCIAL CONNECTION

Thus, behavioral and neural evidence suggest that experiencing generalized shared reality with others—via shared understanding across various contexts—is associated with social connection, which is in turn linked with well‐being. What are the potential mechanisms by which individuals achieve generalized shared reality with others? One possibility is that through the processes of social influence, norms, behaviors, and attitudes spread throughout social networks (Cialdini & Goldstein, 2004; Cialdini & Trost, 1998) and lead to the construction of shared meaning (Baumeister et al., 2018). Social influence pervades human society and has consequently been of great interest in the field of psychology, as evidenced by decades of research and theorizing on the topic (Cialdini & Trost, 1998; Turner, 1991). Findings from classic social psychology experiments suggest that individuals achieve shared understanding with others across various contexts because they are strongly driven by motivations both to socially affiliate and belong (“normative conformity”) and to form accurate perceptions of the world around them and therefore seek information from others to inform their own perceptions in ambiguous situations (“informational conformity”) (Asch, 1956; Deutsch & Gerard, 1955; Sherif, 1935, 1936). For instance, the famous Asch (1951) experiments, which found that a majority of individuals conformed in their judgments about the length of lines to match that of an erroneous majority, provide examples of normative conformity, highlighting that individuals are so strongly driven by motivations to socially affiliate (Baumeister & Leary, 1995) that they will often portray an *ostensible* generalized shared reality with others even at the cost of accuracy (Chen et al., 1996). Furthermore, corroborating theories of informational conformity, classic experiments by Sherif (1935, 1936) showed that groups converge over time to form common norms about ambiguous stimuli, providing insight into how social influence that leads to shared norms can occur rather seamlessly, particularly in unclear situations.

Neuroimaging evidence supports these accounts of social influence. For instance, individuals feel distressed when they find out that they are misaligned with others; brain regions associated with conflict detection are activated when people learn that their own attitudes and opinions are different from their peers' (Klucharev et al., 2009), while changing one's own opinions to conform to peers' opinions is associated with increased activity in the brain's subjective valuation regions (Welborn et al., 2015). These findings provide insight into the biological mechanisms underlying normative conformity, suggesting that the experience of shared understanding is rewarding, whereas lacking shared understanding may trigger a neural “alarm” signal, potentially motivating efforts to conform and reestablish a sense of a shared worldview with others. Accordingly, these processes may support relational motives that promote generalized shared reality across individuals (Higgins et al., 2021). Neural evidence also suggests that social influence can promote genuine and private acceptance of social norms; in one study, social influence modulated brain regions that encode subjective valuation, such that stimuli (i.e., faces) elicited greater valuation‐related brain activity after participants learned that their peers had allegedly rated those stimuli as more attractive than they had, and less valuation‐related brain activity after participants learned that their peers had allegedly rated those stimuli as less attractive than they had (Zaki et al., 2011); these findings provide neurobiological evidence supporting informational conformity and epistemic motives that lead to generalized shared reality, suggesting that individuals genuinely update their own perceptions about the world in light of information from others. Taken together, conformity may be one route by which societies come to have a unified generalized shared reality, arriving at an agreement in beliefs, attitudes, and norms, driven by motivations to belong and to form accurate representations of the world.

As briefly discussed above, people tend to be surrounded by others who share many characteristics as themselves (McPherson et al., 2002). Does similarity drive social connection or does social connection lead to similarity? In other words, are people attracted to people similar to themselves, or do people become more similar to their social ties over time? Evidence suggests a bidirectional effect. For instance, people tend to gravitate toward others who share similar attitudes and traits as themselves, viewing them more favorably than dissimilar others (Byrne, 1961, 1997; Montoya & Horton, 2013), supporting the notion that similarity drives social connection, such that people are more likely to feel socially connected with others who are already similar to themselves. A separate body of research provides empirical evidence that supports the reverse causal direction. Social influence processes (such as those described in the preceding paragraph on conformity) unfold amongst people in one another's immediate social contexts, causing ways of thinking, feeling, and behaving to spread through social networks such that over time, people become similar to those who are close to them in social ties (e.g., friends, friends‐of‐friends) in a diversity of ways (Cacioppo et al., 2009; Christakis & Fowler, 2007; Rosenquist et al., 2011). Future studies can provide further insight into not only the *extent* to which homophily versus social influence contribute to links between shared understanding and social connection, but also the *types* of similarities among close social ties that may result from preexisting similarities versus social influence. Furthermore, it could be fruitful to consider the role of culture in these relationships; for instance, individuals with shared cultural backgrounds interpret signals to others' emotional states more similarly, facilitating increased sensitivity and accuracy in recognizing one another's emotional expressions (Elfenbein & Ambady, 2002). Future work could explore whether people with shared cultural backgrounds would have a greater likelihood of social connection due to alignment in their interpretations of cues to others' apparent emotional states. Future work investigating the relationship between shared reality and non‐affiliative forms of social connection could also be fruitful. For instance, it remains largely unknown whether competitive interactions (where there are both winning and losing parties, such as sports games) lead to shared reality (because individuals were in the same situation) or not (because outcomes differed across individuals and individuals' goals were misaligned with one another).

## 4 APPLICATIONS TO STUDY SOCIAL PHENOMENA

**FIGURE 3:**
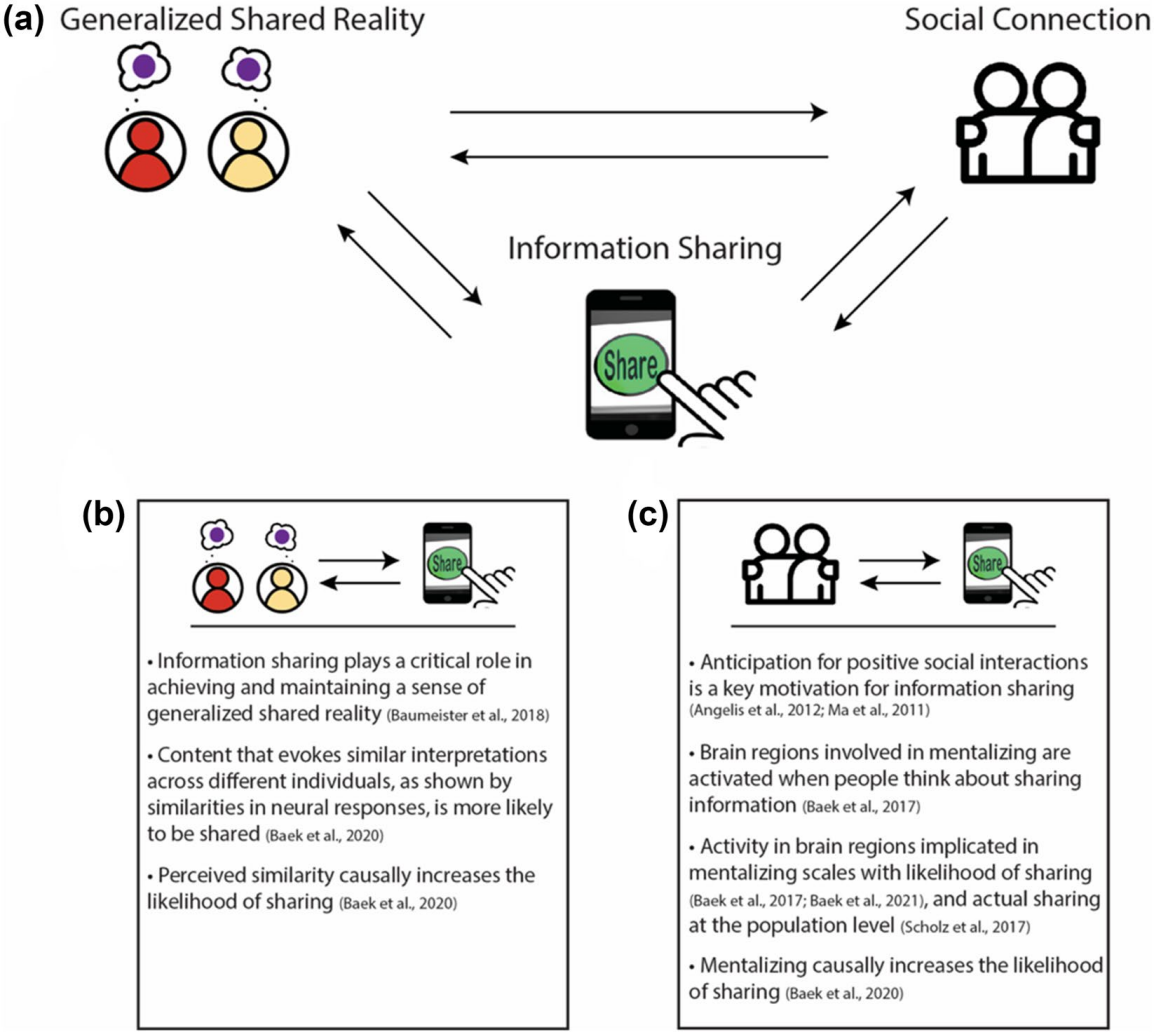
Information sharing, generalized shared reality, and social connection. (a) The associations between generalized shared reality and social connection can be used as a theoretical framework to understand the mechanisms that underlie information sharing. (b) The association between information sharing and generalized shared reality is bidirectional; people share information that reflects shared norms and values of a social group, and this sharing behavior in turn contributes to the further creation and reinforcing of shared realities. (c) The association between information sharing and social connection is bidirectional. Desires to socially connect drive information sharing, and information sharing leads to social interactions, which can result in social bonding

As we have discussed thus far, a large body of literature supports the notion that shared understanding across various contexts is linked to social connection. Emerging research suggests that one ubiquitous human behavior, information sharing, is rooted in both a desire to establish shared understanding with others and can serve to reinforce generalized shared reality more broadly. In this section, we synthesize behavioral and neural studies that corroborate recent theories suggesting that information sharing plays a critical role in the pursuit and creation of generalized shared reality in society (Baumeister et al., 2018), one that helps promote and reflects social connection between individuals (see Figure 3).

Information sharing is an inherently social behavior. The act of sharing information with others supports fundamental human motivations to connect and belong (Baumeister & Leary, 1995; Berger, 2014), and people self‐report anticipation for positive social interactions as a key motivation for sharing information (Angelis et al., 2012; Ma et al., 2011). Recent neuroimaging work corroborates these findings, highlighting that brain regions involved in mentalizing play an important role in information sharing. For instance, such brain regions (e.g., medial prefrontal cortex, precuneus, temporoparietal junction, superior temporal sulcus; Dufour et al., 2013; Frith & Frith, 2003) are activated when people make decisions about sharing information, compared to other types of decisions, and the extent to which information (e.g., news articles) activates these regions is associated with the likelihood of sharing (Baek et al., 2017). Activity in these regions is also positively associated with greater likelihood that online recommendations will propagate (Baek, O'Donnell et al., 2021). Furthermore, a neural model of information virality (“value‐based virality”) posits that social considerations contribute to an overall subjective value signal that represents the expected value of sharing a piece of information; this overall value signal is directly associated with population‐level virality (how often information is actually shared in the real world; Scholz et al., 2017). In other words, the more that a piece of content evokes activity in mentalizing‐related brain regions, the higher the overall value signal of sharing, which is then associated with population‐level sharing behavior (Scholz et al., 2017).

Recent behavioral experiments extend these neural findings to suggest that this relationship between mentalizing and sharing is *causal*, such that mentalizing drives sharing; people are more likely to share information when instructed to think about other people's minds compared to control conditions (Baek, Tamir et al., 2020). As such, people appear to actively consider other people's mental states when considering content to share with others and are motivated to share information to fulfill their needs to socially connect with others.

Thus, the extent to which a piece of content engages the brain's mentalizing regions is associated with both individual and population‐level sharing behavior, and mentalizing increases the likelihood of sharing. Given that experiencing generalized shared reality with others is strongly linked to social connection, one possibility is that these processes drive information sharing; people may share information that they believe that others will also find valuable because by doing so, they reinforce the shared perspectives, attitudes, and beliefs about happenings of the world around them that are already well‐established and agreed upon in their social circles. These effects may then create a positive reinforcing loop; sharing information that reflects the shared worldviews of a social group also contributes to the further creation and reinforcing of generalized shared realities (Figure 3).

Indeed, recent work provides empirical support for the idea that information sharing may be intertwined with motivations to achieve generalized shared reality with others. In one study, brain activity was measured while participants viewed naturalistic videos and provided their likelihood of sharing each video. Similarities in neural responses in regions associated with both low‐level sensory processing and high‐level cognition, including brain regions implicated in mentalizing, were associated with greater sharing likelihood, suggesting that people are more likely to share content that evokes similar interpretations across different individuals in a social group (Baek, Hyon et al., 2020). These relationships were explicitly tested and supported in follow‐up behavioral experiments, which showed that people are more likely to share information when they believe that others in their social circles would share their own viewpoints on the information (Baek, Hyon et al., 2020). Preliminary evidence also suggests that the relationship is causal, such that perceived shared understanding increases the likelihood of sharing (Baek, Hyon et al., 2020). Combined, these findings support the idea that people are motivated to create and reinforce generalized shared reality about the world around them with others in their social circles, and that information sharing may be one way that such generalized shared realities are formed and maintained.

Notably, the mentalizing‐related regions of the brain that have been identified to be important in the information sharing process are part of the broader default mode network (Mars et al., 2012; Meyer et al., 2018; Spunt et al., 2013). As discussed above, the default mode network has been theorized to play a critical role in integrating individuals' internal states with their environment (Yeshurun et al., 2021), and similarities in neural responding in regions of this network have been linked with shared understanding and interpretation of events (Lahnakoski et al., 2014; Nguyen et al., 2019; Yeshurun et al., 2017) and friendship (Parkinson et al., 2018). The implication of these regions in information sharing corroborate theories of information sharing as critical in creating and establishing collective meaning that bind social groups together through shared worldviews (Baumeister et al., 2018). In these ways, information sharing may fulfill both types of motives that have been proposed to underlie generalized shared reality by not only helping people relate to one another interpersonally (“relational motives”), but also to interpret and understand the world around them (“epistemic motives”) (Higgins et al., 2021).

As we uncovered through the example of information sharing, the established associations between shared understanding across various contexts (and thereby generalized shared reality more broadly) and social connection have the potential to be used as a theoretical framework to elucidate mechanisms underlying various social psychological phenomena of interest. For instance, this framework could be used to study the motivations that drive the spread of misinformation, a behavior that has widespread negative consequences (Allcott & Gentzkow, 2017; Kata, 2010). Future research could test whether individuals' motivations to maintain and promote generalized shared reality with others in their social circles may lead them to be less concerned about the accuracy of content before sharing it, further perpetuating beliefs and attitudes based on false information in their communities. This framework can also be applied to uncover features in pro‐health and prosocial messages that are more likely to be successful in persuasion and behavior change, which has the potential for widespread positive outcomes. For instance, one could use tools such as neuroimaging to test whether public service announcements that are likely to be similarly interpreted and understood across individuals in a social group would be more successful in producing message‐congruent behavior change compared to messages that evoke idiosyncratic responses across individuals. Indeed, results from a small body of work provide preliminary support for this idea; effective speeches elicit greater neural similarity compared to ineffective speeches (Schmälzle et al., 2014), and neural similarity scales with real‐world engagement levels of television shows and advertisements beyond individuals' self‐report ratings (Dmochowski et al., 2014). Future work that explicitly tests whether similarly‐interpreted messages are more likely to be successful because they promote generalized shared reality (which is important to social connection and group cohesion) could be particularly fruitful. Another potential future direction is to investigate whether one could promote social connection by increasing neural similarity and correspondingly, shared understanding about various topics; given findings that suggest that lower levels of objective and subjective social connection are linked to idiosyncratic processing of the world (Baek et al., 2022; Baek, Hyon et al., 2021), interventions that promote shared understanding in different contexts, especially in populations who are vulnerable to social isolation, could be particularly fruitful. As these examples highlight, applying the established links between shared understanding and social connection as a framework to study psychosocial phenomena has the potential to advance understanding of the mechanisms that underlie such phenomena. This is a particularly exciting time to explore such questions, as the emergence of interdisciplinary methods that combine approaches from psychology, neuroscience, and network science has the potential to synergistically advance theoretical understanding of the associations between generalized shared reality, social connection, and psychosocial phenomenon that characterize the social world.

## 5 CONCLUSIONS

Recent research that integrates tools from neuroscience with approaches to study social networks corroborates and extends the importance of shared understanding across various contexts—and generalized shared reality more broadly—in social connection. The extent to which individuals show similarities in neural responding with one another is linked to both objective and subjective social connection, such that well‐connected and not‐lonely individuals are similar to one another, whereas less‐connected and highly‐lonely individuals show idiosyncrasy in neural responding that may reflect a lack of generalized shared reality with others. These findings support the notion that sharing one's worldview with social partners is critically related to social connection. Future work that applies this framework to study various psychosocial phenomena may be particularly fruitful in contributing to relevant theories in psychology, neuroscience, and related fields.

## CONFLICT OF INTEREST

Authors declare no conflict of interest.
